# Carbon Nanofiber Membranes Loaded with MXene@g-C_3_N_4_: Preparation and Photocatalytic Property

**DOI:** 10.3390/nano14100896

**Published:** 2024-05-20

**Authors:** Ching-Wen Lou, Meng-Meng Xie, Yan-Dong Yang, Hong-Yang Wang, Zhi-Ke Wang, Lu Zhang, Chien-Teng Hsieh, Li-Yan Liu, Mei-Chen Lin, Ting-Ting Li

**Affiliations:** 1 Innovation Platform of Intelligent and Energy-Saving Textiles, School of Textile Science and Engineering, Tiangong University, Tianjin 300387, China; 2 Department of Bioinformatics and Medical Engineering, Asia University, Taichung 413305, Taiwan; 3 Department of Medical Research, China Medical University Hospital, China Medical University, Taichung 404333, Taiwan; 4 Tianjin Fire Science and Technology Research Institute of MEM, Tianjin 300381, China; 5 Tianjin and Ministry of Education Key Laboratory for Advanced Textile Composite Materials, Tiangong University, Tianjin 300387, China; 6 Department of Fashion Design and Merchandising, Shih Chien University, Kaohsiung 84550, Taiwan; 7 Department of Biomedical Engineering, College of Biomedical Engineering, China Medical University, Taichung 404333, Taiwan

**Keywords:** photocatalytic activity, recyclability, reusable performance, g-C_3_N_4_ nanosheets, MXene nanomaterials

## Abstract

In this study, a Ti_3_C_2_ MXene@g-C_3_N_4_ composite powder (TM-CN) was prepared by the ultrasonic self-assembly method and then loaded onto a carbon nanofiber membrane by the self-assembly properties of MXene for the treatment of organic pollutants in wastewater. The characterization of the TM-CN and the C-TM-CN was conducted via X-ray diffraction (XRD), scanning electron microscopy (SEM), X-ray photoelectron spectroscopy (XPS), and Fourier transform infrared spectrometer (FTIR) to ascertain the successful modification. The organic dye degradation experiments demonstrated that introducing an appropriate amount of Ti_3_C_2_ MXene resulted in the complete degradation of RhB within 60 min, three times the photocatalytic efficiency of a pure g-C_3_N_4_. The C-TM-CN exhibited the stable and outstanding photocatalytic degradation of the RhB solution over a wide range of pH values, indicating the characteristics of the photodegradation of organic pollutants in a wide range of aqueous environments. Furthermore, the results of the cyclic degradation experiments demonstrated that the C-TM-CN composite film maintained a degradation efficiency of over 85% after five cycles, thereby confirming a notable improvement in its cyclic stability. Consequently, the C-TM-CN composite film exhibits excellent photocatalytic performance and is readily recyclable, making it an auspicious eco-friendly material in water environment remediation.

## 1. Introduction

With the development of human society since the first industrial revolution in the 18th century, global production and manufacturing industries have rapidly developed, leading to environmental damage and pollution to human production and living. So far, millions of organic chemicals, particularly organic dyes, drugs, personal care products, and pesticides, have been discovered, synthesized, and produced. Once organic dyes enter into water, it is no longer safe to drink, and it is sometimes difficult to completely treat such contaminated water [1,2]. Organic dyes are common pollutants in the textile dyeing, plastics, and paper industries, and many of them are aromatic compounds with potential carcinogenic and mutagenic effects [3]. Various wastewater treatment techniques have been developed [4,5,6,7,8,9,10]. However, compared with other wastewater treatment technologies, photocatalytic technology does not require secondary pollutants after treatment. Moreover, photocatalytic technology does not require secondary pollutants after treatment and also has a series of advantages such as high efficiency, stability, and easy operation, which has a broad application prospect in energy development and environmental remediation [11]. However, most semiconductor photocatalytic materials typically exist in the form of powders, but powder photocatalysts are often prone to aggregation in water, which reduces their light absorption capacity and utilization efficiency. In addition, powder materials are challenging to recycle and reuse, leading to high application costs, thereby limiting the practical application of semiconductor photocatalysts [12,13]. The photocatalytic membrane developed by combining photocatalysts and membrane materials is one of the most effective methods for solving photocatalysts’ recycling utility.

Since Liu and Cohen predicted, in 1989, that carbon nitride compounds had the potential to become superhard materials [14], research on carbon nitride materials has become increasingly in depth. Graphitic carbon nitride (g-C_3_N_4_, CN) is known as a potential photocatalyst because of its outstanding thermal and chemical stability, non-toxicity, easy preparation, and low cost [15,16,17,18,19,20]. Kroke [21], through density functional theory (DFT) calculations, found that graphitic carbon nitride (g-C_3_N_4_) built by tri-s-triazine units was considered to be the most stable homolog in the environment among various isomers. As a photoelectric catalyst, g-C_3_N_4_ can separate holes and electrons, and its 2.7 eV bandgap enables it to absorb sunlight. However, the photocatalytic activity of g-C_3_N_4_ has intrinsic shortcomings, such as high charge recombination efficiency, inhibiting conductivity, and insufficient surface area caused by self-aggregation [22,23,24,25,26,27]. Researchers have explored many strategies to overcome these intrinsic deficiencies, such as creating new forms, chemical blending, auxiliary catalysis loading, modifying defects, controlling crystal structures, and constructing heterojunctions.

In recent years, two-dimensional transition metal carbides, nitrides, and carbonitrides have developed rapidly and gradually become a research hotspot for photocatalytic material development. MXene, as a new emerging two-dimensional material, has excellent properties such as a typical layered structure, tunable element composition, excellent conductivity, persistent structure stability, adjustable surface functional groups, element abundance, and large surface area, making them a powerful robust candidate material for various applications in photocatalysis [28,29]. As a photocatalyst carrier or auxiliary catalyst, or in combination with other two-dimensional semiconductor materials to improve photocatalytic efficiency, MXene materials can promote light-induced carrier separation by various actions, thereby enhancing the photocatalytic activity of other photocatalysts, such as strongly supporting the homogeneous growth and fine dispersion of other photocatalysts, effectively replacing precious metals, and enhancing the adsorption of reactants. In 2011, the earliest MXene materials discovered were Ti_3_C_2_Tx, followed by the preparation of about 20 different unusual MXene materials. Ti_3_C_2_ was a widely studied MXene material with excellent properties. As a two-dimensional (2D) transition metal carbide, Ti_3_C_2_ MXene has a conductivity of 4600 ± 1100 S cm^−1^ with highly anisotropic carrier mobility, which facilitates the material for electron trapping and transfer [30,31,32]. Secondly, the Ti_3_C_2_ surface has end groups that can provide active sites and also provide strong interfacial solid contact between the co-catalyst and the photocatalyst [33,34,35,36]. In addition, its 2D lamellar structure has a large specific surface area [33] and a suitable figure of merit. These properties are very promising for the co-construction of heterojunction-structured photocatalytic materials with other semiconductor materials, which can improve the photocatalytic performance of single catalysts [37]. Under visible light irradiation, the electron–hole in a photocatalytic reaction undergoes a jump to form an electron (e^−^) and hole (h^+^) in its conduction and valence bands, respectively [35]. Appropriately designed heterojunction photocatalysts were shown to be capable of higher photocatalytic activity because of the spatial separation of electron–hole pairs generated by photoluminescence [36]. Since electrons have reducing power and holes have oxidizing power, they can react with OH-, O_2_, and H_2_O adsorbed on the surface of the material to form radicals with strong oxidizing activity, such as -OH, -O_2_^−^, -OOH, and other radicals with strong oxidizing activity [37]. Combining MXene, an excellent metal conductive material, with g-C_3_N_4_ can effectively provide shorter electron transport channels and accelerate the rapid movement of electrons [38].

To begin with, an ultrasonic self-assembly measure was applied to form TM-CN in this study, and TM-CN was loaded over carbon fibers via the Ti_3_C_2_ MXene self-assembly films. The organic dye degradation test indicates that an appropriate amount of Ti_3_C_2_ MXene can significantly improve the photocatalytic activity of g-C_3_N_4_. After calculations, g-C_3_N_4_ and Mxene have a staggered energy band structure, promoting the rapid separation of photogenerated electron–hole pairs, reducing the degree of electron–hole complexation, and improving the photocatalytic degradation of pollutants. Surface-loaded TM-CN carbon nanofiber membranes exhibited excellent photocatalytic degradation of organic dyes under various pH conditions. Moreover, powder photocatalysts loaded on carbon nanofiber membranes improved the problem of the difficult recycling of powder materials, and C-TM-CN maintained excellent photocatalytic effects in five photocatalytic cycle degradation experiments.

## 2. Experiments and Methods

### 2.1. Experimental Materials

Polyvinyl pyrrolidone, isopropyl alcohol (IPA), and LiF were procured from Shanghai Aladdin Bio-Chem Technology Co., Ltd. (Shanghai, China) N,N-Dimethylformamide (DMF) was obtained from Tianjin Bohua Chemical Reagent Co., Ltd. (Tianjin, China) Carbamide and HCl were obtained from Tianjin Fengchuan Chemical Reagent Co., Ltd. (Tianjin, China) Polyacrylonitrile was purchased from Spectrum Chemical (Shanghai) Co., Ltd. (Shanghai, China) Rhodamine B (RhB), methylene blue (MB), and methyl Orange (MO) were procured from Beijing Solarbio Science & Technology Co., Ltd. (Beijing, China) Benzoquinon, ethylenediaminetetraacetic acid disodium salt (EDTA-2Na), and NaOH were obtained from Tianjin Kermel Chemical Reagent Co., Ltd. (Tianjin, China) Ti_3_AlC_2_ was obtained from Ningbo Jinlei Nano Material Technology Co., Ltd. (Ningbo, China) All chemicals used in this study without particular states were of analytical grade and used as received. All solutions were prepared with deionized water.

### 2.2. Preparation of g-C_3_N_4_

This study chose a conventional thermal polymerization method to prepare the g-C_3_N_4_ powder. Melamine powder was chosen as the raw material, and it was placed in a covered ceramic crucible and heated in a muffle furnace to 550 °C (with a ramp rate of 3 °C min^−1^) and held for 4 h to synthesize bulk g-C_3_N_4_. After this, the bulk g-C_3_N_4_ was ground in a mortar and pestle to produce g-C_3_N_4_ powder.

### 2.3. Preparation of Ti_3_C_2_ MXenes

**Figure 1:**
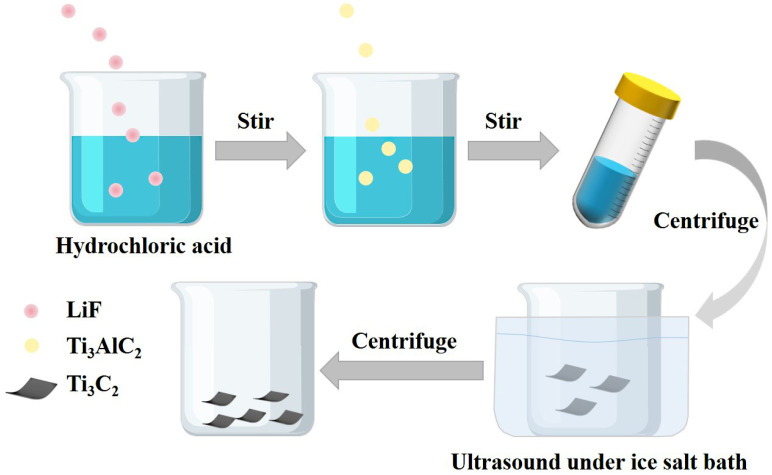
Preparation process of Ti_3_C_2_ MXene.

Firstly, 1 g lithium fluoride (LiF) powders were dispersed in 20 mL of 9 M hydrochloric acid and then mixed for 30 min at room temperature, forming a LiF/HCl mixture. Afterwards, 1 g of Ti_3_AlC_2_ was added to the LiF/HCl mixture for another 24 h, blending at 40 °C to remove the Al etch. The final suspension was repeatedly rinsed with deionized water and underwent centrifugation at 4000 rpm for five minutes until the pH was 6. At last, the black swelling clay-like sediment mounted in an ice salt bath underwent consecutive ultrasonic treatment for 2 h and then centrifugation for 1 h. The supernatant from the final centrifugation was used to ensure that Ti_3_C_2_T_x_ had separated (See Figure 1).

### 2.4. Preparation of TM-CN

To begin with, 10 mg g-C_3_N_4_ was dispersed in 100 mL of deionized water and then ultrasonic processed for 2 h, after which 100 μg of the resulting solution was removed and mixed with 10 mL of deionized water for another 15 min of ultrasonic treatment to attain an even distribution. Ti_3_C_2_ solution was slowly dripped into the g-C_3_N_4_ suspension, and the mixture was ultrasonically processed for 4 h, forming TM-CN.

### 2.5. Preparation of TM-CN-Loaded Carbon Fibers

**Figure 2:**
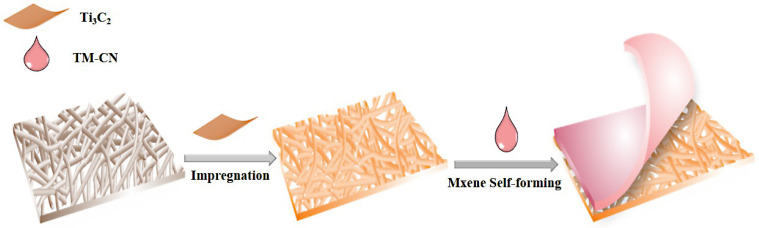
Preparation process of C-TM-CN.

To begin with, 1 mg of TM-CN (powders) was dispersed in 4 mL of deionized water, after which the carbon nanofiber membrane was soaked in a 15 μg mL^−1^ Ti_3_C_2_ solution for fifteen minutes. The membrane was removed and baked in an oven until the surface became viscous. Next, the well-distributed TM-CN suspension was dripped over the surface of the composite membranes. The impregnated composite membranes were baked in an oven for 1 h, forming the C-TM-CN (See Figure 2).

### 2.6. Characterization

A scanning electron microscope (HITACHI S4800, Japan Hitachi Limited, Tokyo, Japan) was used to study the micromorphology, size, and composition based on SEM images. The XRD measurement (D8 Discover, Bruker company, Bremen, Germany) was to observe the crystal structure, while XPS (D8 Discover, Bruker company, Germany) was used to measure the constituent elements and valence state. Moreover, a UV–visible spectrometer (UV2600, Ltd. echcomp Science and Technology, Shanghai, China) was used to measure the photo absorption of catalysts. The electrochemical workstation (CHI 660E, Shanghai Chen Hua Electric Furnace Co., Ltd., Shanghai, China) was utilized to measure the photocurrent response and electrochemical impedance of samples.

### 2.7. Photocatalytic Property Characterizations of C-TM-CN

Rhodamine B (RhB) serves as the target pollutant and, as such, examines the photocatalytic properties of the C-TM-CN and the TM-CN. This measurement involved the main reactive species in the photocatalytic response system. Before the dark reactions, 1 mM of isopropyl alcohol (IPA), 1 mM of potassium iodate (KIO_3_), and 1 mM of ethylenediaminetetraacetic acid disodium salt (EDTA-2Na) were added to RhB (50 mL, 10 mg L^−1^) to capture ·OH, ·O_2_^−^, and h^+^ separately. After the 30 min dark reaction, the adsorption equilibrium between the photocatalyst and the pollutant was attained, followed by the conduction of the photocatalytic reaction. The suspension was removed every other (a certain length of) time, and the ultraviolet–visible spectrophotometer (UV2600 Ltd. echcomp Science and Technology, Shanghai, China) measured the absorbance of RhB, thereby computing the photocatalytic degradation efficiency. Next, the C-TM-CN was the photocatalyst in the photocatalytic cyclic decomposition test. In each cycle, forceps were used to remove the membrane that was to be rinsed with deionized water to remove the residual dye, and samples were then baked in an oven at 60 °C for 2 h. Finally, the bake-dried C-TM-CN was once again added to the RhB solution to repeat the next cycle of the photocatalytic decomposition experiment.

## 3. Results and Discussion

### 3.1. SEM Analysis

**Figure 3:**
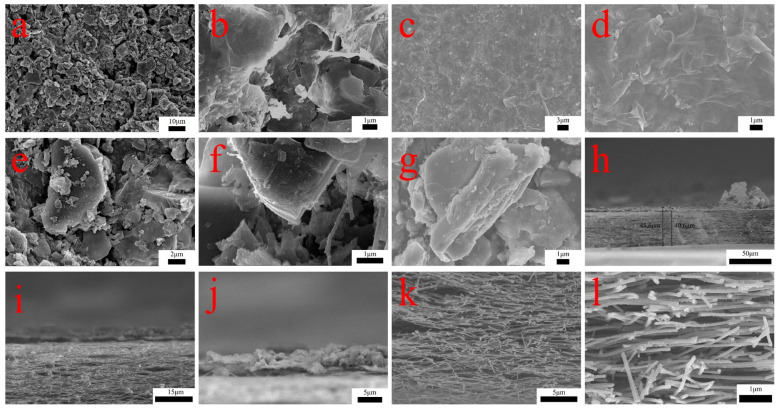
SEM image of C-TM-CN nanofiber composite membrane (**a**–**h**) surface, (**i**–**l**) cross-section.

The micromorphology of C-TM-CN nanofiber composite membranes was observed using scanning electron microscopy (SEM). Figure 3a clearly shows that Ti_3_C_2_ MXene sheets are irregular lumps of two-dimensional nanosheets and that Ti_3_C_2_ MXene sheets and g-C_3_N_4_ particles were bonded. Figure 3a–h present the SEM images of C-TM-CN nanofiber composite membranes, in which Ti_3_C_2_ MXene sheets were self-built membranes, thus successfully forming the membrane structure that covered and adhered to carbon nanofibers concurrently. The TM-CN particles were also successfully formed with the Ti_3_C_2_ MXene sheets during the membrane formation process. The carbon nanofiber membranes and the C-TM-CN thickness are 40.6 μm and 45.6 μm, respectively. Figure 3i–l illustrate the cross-sectional view of the C-TM-CN, wherein the dense Ti_3_C_2_ MXene sheets are observed covering the carbon nanofibers. Evenly distributed g-C_3_N_4_ nanoparticles populate the interior of the carbon nanofiber membranes.

### 3.2. XRD Pattern Analysis and FTIR Spectra Analysis

**Figure 4:**
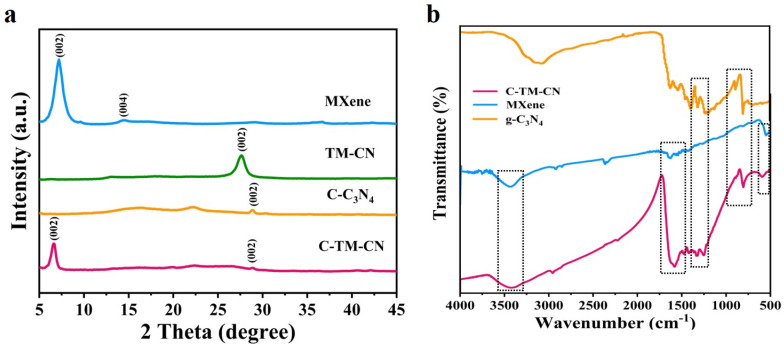
XRD (**a**) pattern and FTIR (**b**) spectra of C-TM-CN, TM-CN, C-C_3_N_4_, C-NM, and Ti_3_C_2_ MXene.

Figure 4a shows the XRD pattern of Ti_3_C_2_, g-C_3_N_4_, and the TM-CN. Based on the XRD patterns, carbon fiber nanofiber membranes (C-NM) exhibit a strong diffraction peak that corresponds to the crystal plane (002). After the loading of Ti_3_C_2_, the peak (002) shifts toward a higher angle, which indicates that the two molecules are strongly interacted, which in turn affects the overlapped distance of g-C_3_N_4_. The findings prove that the heterojunction is formed between Ti_3_C_2_ and g-C_3_N_4_. In addition, there is a strong absorption peak present at 27.7° according to the X-ray powder diffraction pattern of the C-TM-CN. Meanwhile, C-M-C_3_N_4_ nanofiber membranes exhibit diffraction peaks that separately correspond to carbon fiber (22°), g-C_3_N_4_ (27.4°), and Ti_3_C_2_ (5.0°). In Figure 4a, two prominent diffraction peaks were detected at 13.2° and 27.6°, which were indexed to the (100) plane, corresponding to the inplane packing of heptazine units, and the (200) plane, resulting from the characteristic stacking of conjugated aromatics, respectively [39]. After combination with the MXene and carbon nanofiber membrane, the characteristic diffraction peaks of TM-CN and C-TM-CN appeared at the same positions, indicating that the typical layered structure of polymeric g-C_3_N_4_ was well preserved. In addition, the C-TM-CN showed the characteristic diffraction peaks of graphene-like carbon nitride, which was derived from the MXene. Moreover, the typical peak for the Mxene material revealed the successful preparation of Ti_3_C_2_ material and removal of Al layers in the Ti_3_AlC_2_ [40,41,42]. To sum up, Ti_3_C_2_ and g-C_3_N_4_ generate a heterojunction that successfully grows over carbon fiber matrices.

Figure 4b shows the FTIR spectrogram for g-C_3_N_4_, Ti_3_C_2_, and C-TM-CN nanofiber composite membranes. C-TM-CN nanofiber composite membranes exhibit a broader absorption band at 1200– 1800cm^−1^, which corresponds to the C(sp^3^)-N and C(sp^2^)=N in the CN heterocycle of g-C_3_N_4_, but the broader absorption band shifts to the left compared to the CN heterocycle of g-C_3_N_4_. The presence of peaks at 810 cm^−1^ can be attributed to the 3-s-Triazine unit characteristic peak of g-C3N4 [18,43]. The peaks in the range 1135–1629 cm^−1^ were ascribed to the stretching vibrations of heterocycles (C–N/C=N). Furthermore, the broad peak at around 3137 cm^−1^ was assigned to uncondensed terminal amino groups [44,45,46]. This indicated that g-C_3_N_4_ nanomaterials were successfully synthesized. Moreover, the Fourier transform infrared spectroscopy analysis proves the formation of g-C_3_N_4_ over nanofiber composite membranes. As shown in Figure 4b, the firm peaks at 3435 cm^−1^ and 1624 cm^−1^ in the FTIR spectrum of MXene were assigned to the stretching vibrations of the OH and C=O groups, the peak at 620 cm^−1^ belongs to Ti-O group, and the peak at 1432 cm^−1^ was attributed to O-H group bending [47]. As a result, through the self-build membrane capacity of Ti_3_C_2_ MXenes, TM-CN powders are successfully loaded over the C-TM-CN nanofiber composite membranes.

### 3.3. XPS Analysis

**Figure 5:**
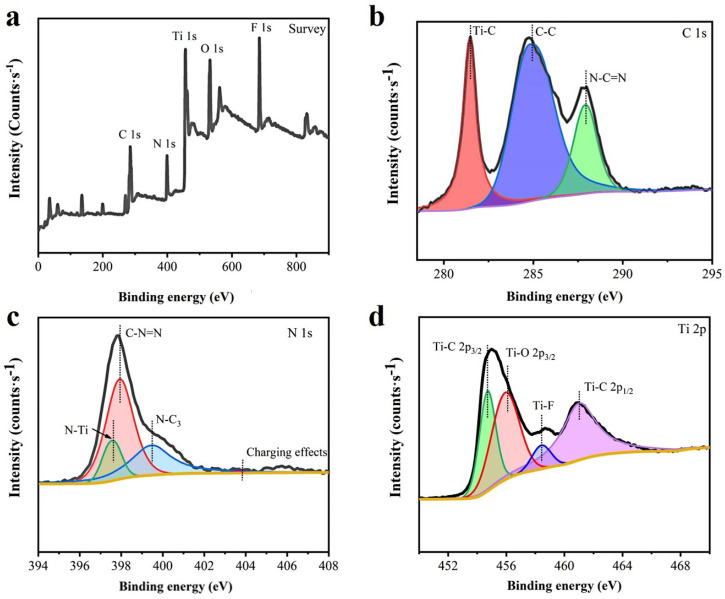
XPS spectra: (**a**) the survey scan of C-TM-CN nanofiber composite membrane; (**b**) C 1s of C-TM-CN nanofiber composite membrane; (**c**) N 1s of C-TM-CN nanofiber composite membrane; and (**d**) Ti 2p of C-TM-CN nanofiber composite membrane.

XPS is used to characterize the components of C-TM-CN nanofiber composite membranes. In Figure 5a, the signature peaks of N, C, and Ti in the C-TM-CN are observed, suggesting the presence of the four elements above in the C-TM-CN. As for the C 1s in Figure 5b, 284.8 eV, 288.1 eV, and 281.5 eV appear as three corresponding binding energy peaks, which represent the interaction between sp^2^ C and Ti-C from the adsorb carbon species of (C-C) and N-C=N, respectively [48,49]. As for the N 1s XPS spectra in Figure 5c, the four binding energy peaks at 397.6 eV, 398.1 eV, and 399.2 eV correspond to N-Ti, C-N=C, and N-C_3_. A new Ti-N peak indicates the heterostructure between the two components. In addition, the peak at 404.1 eV is attributed to the charging effect of the heterocycle [19]. As for the Ti 2p XPS spectra of C-TM-CN in Figure 5d, the peaks of Ti-C 2p_3/2_, Ti-C 2p_1/2_, and Ti-O 2p_3/2_ represent the binding energy at 455.6 eV, 461.1 eV, and 456.6 eV, respectively. In the meanwhile, the binding energy at 458.4 eV is ascribed to Ti-F [20]. The preparation of C-TM-CN was confirmed to be complete based on XPS analyses.

### 3.4. UV–Vis Diffuse Reflection and PL Photoluminescence

**Figure 6:**
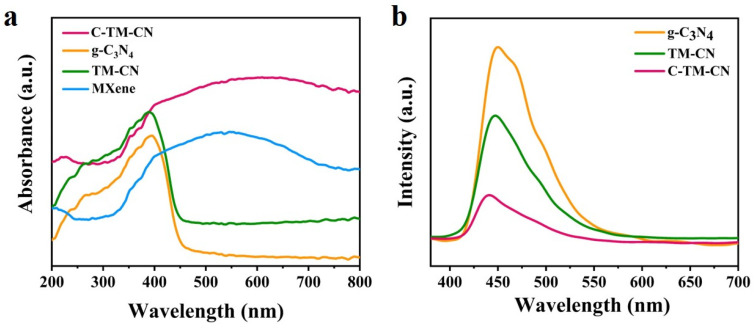
(**a**) UV–Vis diffuse reflectance spectra (DRS) and (**b**) photoluminescence spectra of Ti_3_C_2_, g-C_3_N_4_, TM-CN, and C-TM-CN.

The UV–Vis diffuse reflectance spectra of TM-CN, g-C_3_N_4_, Ti_3_C_2_, and C-TM-CN were examined for photo absorption, as illustrated in Figure 6a. The g-C_3_N_4_ exhibited a powerful absorption spectrum at 460 nm. Similarly, TM-CN exhibited an intense absorption spectrum at 450 nm. Compared to pure g-C_3_N_4_, TM-CN has an absorption margin that blue shifts, which can be attributed to the heterostructure caused by Ti_3_C_2_ and g-C_3_N_4_. In addition, Ti_3_C_2_ MXene has a strong light absorption capability, and its solar spectral response ranges from UV to visible [50,51]. Thus, the loading of Ti_3_C_2_ MXene significantly extends the light absorption range of the C-TM-CN so that it can utilize most of the light in the 200–800 nm range. In summary, the C-TM-CN is a photocatalytic composite membrane with visible light responsive properties.

**Figure 7:**
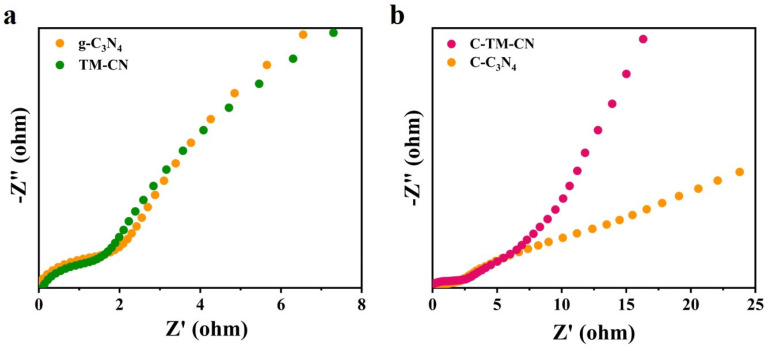
The EIS Nyquist plots of (**a**) g-C_3_N_4_ and TM-CN, (**b**) g-C_3_N_4_, C-C_3_N_4_, and C-TM-CN.

**Figure 8:**
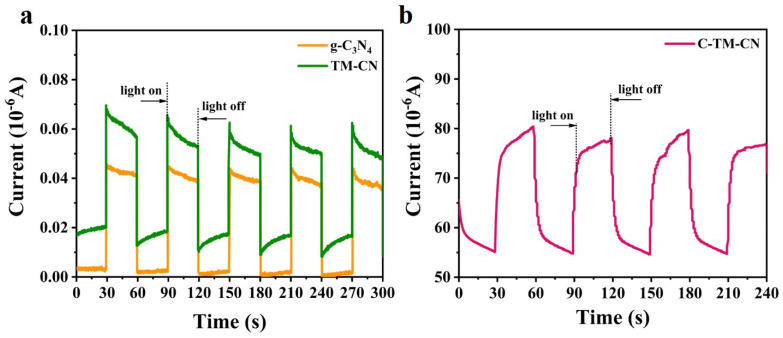
Photocurrent response curves of (**a**) g-C_3_N_4_ and TMCN as well as (**b**) C-TM-CN nanofiber composite membranes.

In the meantime, photoluminescence (PL) spectra are measured to assess the properties of the g-C_3_N_4_, TM-CN, and C-TM-CN nanofiber composite membranes. Photoluminescence (PL) spectroscopy has been regarded as an effective tool for revealing the separation and recombination rates of photogenerated charge carriers in excited photocatalysts. Figure 6b illustrates that the three groups exhibit a photoluminescence range of 400–550 nm. The main peak in the PL spectra of g-C_3_N_4_, TM-CN, and C-TM-CN (Figure 6a) appeared at 450 nm. Still, the photoelectric emission peak of g-C_3_N_4_ was higher than that of TM-CN, indicating that the photogenerated electron–hole complexation rate of CM-TN after the addition of Ti_3_C_2_ MXene material was lower compared to that of g-C_3_N_4_. This result is the same as that shown by the photocurrent results (Figure 7a) and electrochemical impedance response (Figure 8a), which proved that the addition of Ti_3_C_2_ MXene is tightly bonded to g-C_3_N_4_ because of electrostatic interaction to form a practical interface. Moreover, the peak value of the C-TM-CN is significantly lower, indicating that it has the lowest photoluminescence intensity compared to g-C_3_N_4_ and TM-CN.

### 3.5. Electrochemical Impedance

Electrochemical impedance spectroscopy (EIS) is a valuable technique for studying the electrical behavior of photocatalytic systems. It is commonly employed to assess the electron–hole pairs’ transport capacity of photocatalysts. The smaller the arc radius, the smaller the transfer resistance of photogenerated electron-hole pairs enabling the faster surface charge velocity transfer [52,53]. Comparison of the fitted EIS Nyquist plots in Figures S1 and S2 shows that the g-C_3_N_4_ impedance value is smaller than the TM-CN impedance value [54], indicating that the TM-CN has a stronger conductivity, the direct photogenerated electron–hole separation becomes more efficient, and the interfacial charge separation rate is accelerated. Compared to other samples, the C-TM-CN has the smallest impedance arc radius (see Figure 7 and Figure S3). This indicates that the electrons generated by the C-TM-CN nanofibrous membrane can be transported rapidly, promoting the effective separation of the photogenerated electron–hole pairs and prolonging the existence of the photogenerated electron–hole pairs. A higher separation efficiency promotes the photocatalytic activity of the C-TM-CN.

### 3.6. Photocurrent Response

Figure 8a shows the photocurrent response of the TM-CN and g-C_3_N_4_. During the experiment, the assembly is mounted in a camera obscura with an Xe lamp as the light source turned on and off every thirty seconds. The primary voltage was set to 0.5 V. According to the image, under dark conduction, the g-C_3_N_4_ has generated a weak photocurrent of 0.003 μA, and the TM-CN has generated a photocurrent of 0.02 μA. Under light conduction, the TM-CN group has a photocurrent that soars and efficiently arrives at a stable value of about 0.069 μA, which is significantly greater than the g-C_3_N_4_ (0.048 μA). With the Xe lamp being turned off, the dark current rapidly descends to a stable value. Figure 8b shows the photocurrent response of C-TM-CN; with the Xe lamp off, the C-TM-CN generated a photocurrent of approximately 55 μA. With exposure to light, the photocurrent of the C-TM-CN groups swiftly increased to reach a stable value of 75.5 μA. The photocurrent response cycles were repeated three times to ensure the stability of the photocurrent response. The results suggested that the C-TM-CN obtained a better separation efficiency of photogenerated electron–holes because of the electrical conductivity of carbon fibers and Ti_3_C_2_ MXene.

### 3.7. Photocatalytic Properties and Mechanism

**Figure 9:**
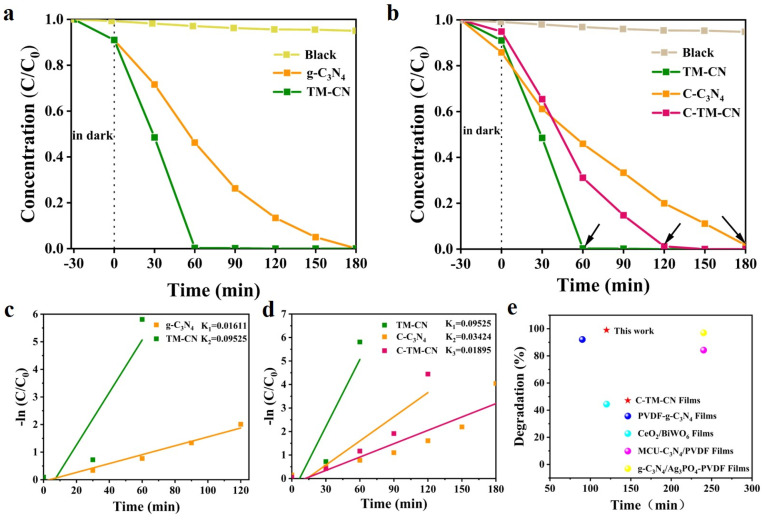
Photocatalytic degradation (**a**) efficiency and (**b**) rate constants of g-C_3_N_4_ and TM-CN on RhB. Photocatalytic degradation (**c**) efficiency and (**d**) rate constants of TM-CN, C-C_3_N_4,_ and C-TM-CN on RhB. (**e**) Comparison of the performance of different photocatalytic materials for RhB degradation.

To investigate the photocatalytic performance of the samples against organic dyes, the photocatalytic degradation of RhB by several materials under visible light conditions was tested. Figure 9a shows that TM-CN reaches 100% degradation efficiency after 60 min, whereas g-C_3_N_4_ takes 180 min to perform the same task, which proves that the photocatalytic performance of TM-CN was greatly improved. Figure 9b illustrates that the photocatalytic degradation constant of TM-CN is 5.91 times higher than that of g-C_3_N_4_. When TM-CN was attached to the carbon nanofiber membrane, the removal rate of 99% was still achieved after 120 min of sunlight irradiation. Compared to the carbon nanofiber membrane loaded with g-C_3_N_4_ (C-C_3_N_4_), the photocatalytic effect of TM-CN exhibited a notable advantage. The photodegradation of RhB is achieved by two competing processes [55]: deethylation at the N-site and disruption of the conjugated structure. Under sun light irradiation, the photocatalyst produces active substances ·OH,·O_2_^−^ and h^+^, which first attack the central carbon atom in the structure of RhB, further decolorize and degrade the dye molecule through the process of deethylation at the N-site and gradually break its conjugated structure, and finally completely mineralize RhB into CO_2_ and H_2_O. It was also observed that the RhB solution became colorless and transparent when the degradation rate reached 99% by TM-CN.

As illustrated in Figure 9e, this study demonstrates a comparable performance to other studies on preparing photocatalytic membrane materials [56,57,58]. The C-TM-CN achieved a superior degradation effect in a shorter period of time than PVDF membrane materials with the addition of pure g-C_3_N_4_ and composite MCU-g-C_3_N_4_ and g-C_3_N_4_/Ag_3_PO_4_ photocatalysts. In addition, the carbon nanofiber membrane used in this work is more environmentally friendly compared to the PVDF membrane. Compared with another CeO_2_/BiWO_6_ membrane, C-TM-CN, with the addition of the TM-CN heterostructured photocatalyst, has a great advantage for the degradation of RhB in an aqueous environment by visible light irradiation.

**Figure 10:**
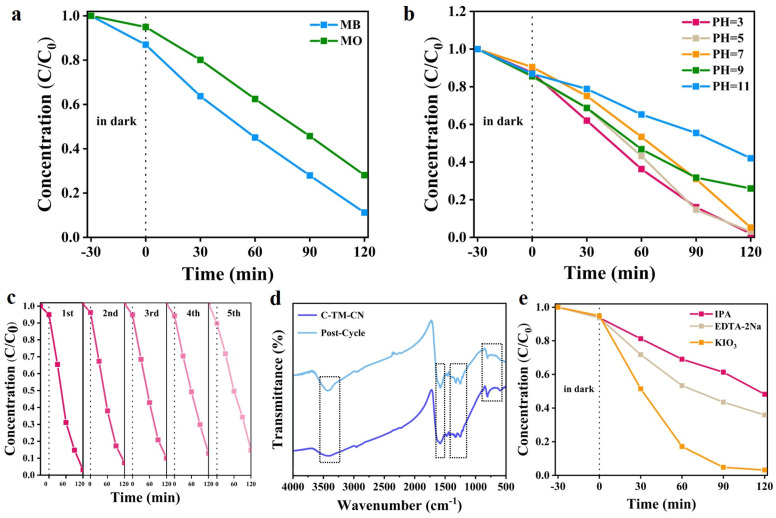
Under visible light irradiation, the photocatalytic degradation of C-TM-CN nanofiber composite membranes’ (**a**) plot of MB solution and MO solution, (**b**) plot of concentration change in degraded RhB under different pH conditions, (**c**) plot of photocatalytic cycle of RhB, (**d**) FTIR spectroscopy before and after photocatalytic degradation, and (**e**) plot of photocatalytic performance of RhB about scavenger.

The C-TM-CN was also tested for the photocatalytic degradation of two organic dyes, methylene blue (MB) and methyl orange (MO). Figure 10a illustrates that the photocatalytic degradation efficiencies of C-TM-CN for MB and MO were 90% and 73%, respectively, after 120 min of light irradiation. This indicated that C-TM-CN has excellent photocatalytic degradation effects on various organic dyes. Furthermore, the photocatalytic degradation of RhB by C-TM-CN was tested under different pH conditions (3–11). Among these, the highest photocatalytic degradation efficiency was exhibited by C-TM-CN at pH 3, as shown in Figure 10b. This is because superoxide, the principal active substance in the photocatalytic degradation reaction, is highly effective in degrading RhB under acidic conditions. Furthermore, studies have demonstrated that the acid effect plays a positive role in the photocatalytic degradation of RhB [59,60,61,62,63]. In contrast, the photocatalytic activity was significantly reduced when the pH was at 11, which may be attributed to electrostatic repulsion. In conclusion, the C-TM-CN has a broad range of application conditions and is suitable for acidic and alkaline wastewater treatment processes.

The stability of the C-TM-CN is another crucial point in the photodegradation of dyeing wastewater. Accordingly, the C-TM-CN was examined for stability for practical use via the five cycles of photodegrading RhB with exposure to visible light, as in Figure 10c. Notably, the C-TM-CN is easily transferred and recycled. In every cycle, the photodegradation against RhB retains greater than 85%, which suggests that the C-TM-CN does not exhibit distinct photocatalytic activity loss. Hence, the prepared C-TM-CN features excellent recycling and photodegradation cycles, which address the problems of powder catalysts, as in the difficulty of recycling and ease of agglomeration while preventing the powders from causing secondary pollution to the water environment. In addition, the chemical composition of C-TM-CN before and after the photocatalytic experiment was characterized by FTIR. As shown in Figure 10d, it can be seen that the samples have absorption peaks belonging to MXene and g-C_3_N_4_ before and after cycling, which proved that the material has good chemical stability during the long cycling test [64].

To study the photocatalytic reaction mechanism of the C-TM-CN, a quenching experiment is conducted as related to the primary active matter in the photodegradation response system (see Figure 10e). As the photocatalyst, the C-TM-CN is used for photocatalytic degradation against the RhB solution. Next, 1 mM EDTA-2Na, 1 mM (KIO_3_) potassium iodate, or 1 mM isopropyl alcohol (IPA) is individually added to the RhB solution as the scavengers to remove H^+^,·O_2_^−^, and ·OH. Exposed to a 500 W Xe lamp, the photocatalytic degradation against the RhB solution of samples is evaluated. Figure 10d exhibits the trapping experiment results for the reactive species. With isopropyl alcohol (IPA) as a trapping agent, the photocatalytic degradation efficiency is reduced to 51.79%, indicating a significant drop in the degradation efficiency. Notably, with KIO_3_ as a trapping agent, the RhB solution exhibits a remarkably reduced absorbance at a maximal light-absorbed wavelength of 552 nm. However, the solution discolors from reddish-purple to yellow, instead of from reddish-purple to transparent. Because the superoxide radical is restricted, the RhB solution fails to have a complete degradation. Similarly, the ethyl group of the benzene nucleus is only partially oxidized rather than wholly oxidized. When EDTA-2Na is used as a trapping agent, the result is the same as the phenomenon with KIO_3_. Therefore, superoxide radicals and cavities interact to a certain extent in the system. To summarize, ·OH, ·O_2_^−^, and H^+^ participate in photocatalytic reactions. Superoxide radicals have a certain service life and can diffuse freely, suggesting that vicarious contact is also greatly involved with photocatalytic degradation.

**Figure 11:**
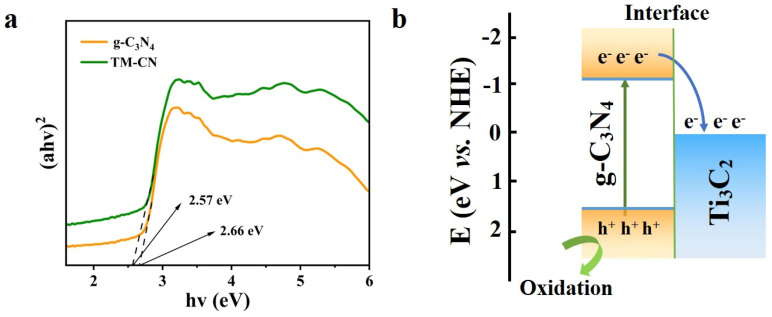
(**a**) The band gap diagram of g-C_3_N_4_, TM-CN, and (**b**) relative energy band positions and charge transfer mechanism between MXene and g-C_3_N_4_.

The excellent organic dye removal performance of the C-TM-CN nanofiber composite membrane is mainly attributed to the enhancement in the light absorption performance by the heterostructure. The close contact between MXene and g-C_3_N_4_ promoted the rapid transfer and separation of the photoelectron–hole pair, which enhanced the photocatalytic performance. Based on Figure 6a’s UV–Vis diffuse reflectance spectra (DRS), the Eg of the g-C_3_N_4_ and TM-CN were calculated as 2.66 eV and 2.57 Ev, used Equation (1), and made into Figure 11a. The Eg of TM-CN after adding MXene was reduced compared to that of pure g-C_3_N_4_, which implied a superior utilization of light absorption by TM-CN. Therefore, the TM-CN photocatalytic degradation efficiency of RhB under visible light irradiation was significantly improved compared to pure g-C_3_N_4_ (see Figure 9a). The positions of the conduction and valence bands of the g-C_3_N_4_ were calculated according to Equations (2) and (3). The ECB position g-C_3_N_4_ was calculated as −1.055 eV. Then, the EVB corresponds to 1.515 eV. Thus, the band structure [48,65] of g-C_3_N_4_ after contact with MXene is shown in Figure 11b. The results showed a staggered energy band structure between the g-C_3_N_4_ and Ti_3_C_2_. Consequently, the carbon fiber membrane C-TM-CN loaded with TM-CN had more muscular photocatalytic activity and higher efficiency in degrading RhB than the carbon fiber membrane C-C_3_N_4_ loaded with pure g-C_3_N_4_ (see Figure 9b).

(αhv)^1/n^ = A(hv − Eg) (1)

E_CB_ = X − E_C_ − 1/2Eg (2)

E_VB_ = Eg+ E_CB_ (3)

Under visible light irradiation, the electron and hole pair jumps on the material’s surface, forming photoelectron–hole pairs in its conduction and valence bands, respectively. Since electrons are reductive and holes are oxidative, they can react with OH-, O_2,_ and H_2_O adsorbed on the surface of the C-TM-CN nanofiber membrane to generate free radicals with oxidative solid activity. These free radicals can be directly oxidized to remove organic dye molecules in solution. However, if the electron transfer efficiency is low and the photoelectron–hole pair complexation efficiency is high, it will reduce the photocatalytic degradation of pollutants. In contrast, the tight binding of MXene and g-C_3_N_4_ provided more efficient electron transfer conditions, accelerating the photocatalytic degradation of organic dyes.

## 4. Conclusions

In this study, TM-CN nanoparticles and C-TM-CN nanofiber composite membranes loaded with TM-CN nanoparticles were successfully prepared. According to the analysis of XRD, SEM, and XPS, the TM-CN nanoparticles were uniformly distributed on the carbon nanofiber membranes. The test results showed that the TM-CN nanoparticles and C-TM-CN nanofiber composite membranes exhibited excellent photocatalytic performance. The C-TM-CN showed the relatively photocatalytic degradation of organic dyes at all conditions of 3–11 pH. Especially, the C-TM-CN exhibited 99% photocatalytic degradation efficiency for RhB under simulated sunlight conditions when the pH value was 3.0. In addition, the C-TM-CN nanofiber composite membrane was tested for the cycling of photocatalytic RhB solution, and it still maintained a photocatalytic degradation rate higher than 85% after five cycling experiments. The quenching experiments confirmed the photocatalytic activity of the C-TM-CN nanofiber composite membrane for RhB because of its combined effect of ·OH^−^, ·O_2_^−^, and H^+^. Therefore, the C-TM-CN nanofiber composite membrane can be reused and easily recycled, making it a promising environmental material in the field of water environment remediation.

## Data Availability

Data are contained within the article.

## Supplementary Materials

The following supporting information can be downloaded at: https://www.mdpi.com/article/10.3390/nano14100896/s1, Figure S1: The Nyquist plots and corresponding circuits of g-C3N4; Figure S2: The Nyquist plots and corresponding circuits of TM-CN; Figure S3: The Nyquist plots and corresponding circuits of C-TM-CN.

## References

[1] Rai H.S., Bhattacharyya M.S., Singh J., Bansal T.K., Vats P., Banerjee U.C. (2005). Removal of dyes from the effluent of textile and dyestuff manufacturing industry: A review of emerging techniques with reference to biological treatment. Crit. Rev. Environ. Sci. Technol..

[2] Santhosh C., Velmurugan V., Jacob G., Jeong S.K., Grace A.N., Bhatnagar A. (2016). Role of nanomaterials in water treatment applications: A review. Chem. Eng. J..

[3] Forgacs E., Cserháti T., Oros G. (2004). Removal of synthetic dyes from wastewaters: A review. Environ. Int..

[4] Ali I., Khan T.A., Asim M. (2011). Removal of Arsenic from Water by Electrocoagulation and Electrodialysis Techniques. Sep. Purif. Rev..

[5] Beluci N., Mateus G., Miyashiro C.S., Homem N., Gomes R.G., Fagundes-Klen M.R., Bergamasco R., Vieira A.M.S. (2019). Hybrid treatment of coagulation/flocculation process followed by ultrafiltration in TIO2-modified membranes to improve the removal of reactive black 5 dye. Sci. Total Environ..

[6] Dai S.-Q., Jiang Y.-Y., Wang T., Wu L.-G., Yu X.-Y., Lin J.-Z., Shi S.-X. (2016). Enhanced performance of polyimide hybrid membranes for benzene separation by incorporating three-dimensional silver–graphene oxide. J. Colloid Interface Sci..

[7] Gupta V.K., Agarwal S., Saleh T.A. (2011). Synthesis and characterization of alumina-coated carbon nanotubes and their application for lead removal. J. Hazard. Mater..

[8] Gupta V., Nayak A. (2012). Cadmium removal and recovery from aqueous solutions by novel adsorbents prepared from orange peel and Fe_2_O_3_ nanoparticles. Chem. Eng. J..

[9] Homem N.C., Beluci N.d.C.L., Amorim S., Reis R., Vieira A.M.S., Vieira M.F., Bergamasco R., Amorim M.T.P. (2019). Surface modification of a polyethersulfone microfiltration membrane with graphene oxide for reactive dyes removal. Appl. Surf. Sci..

[10] Shao F., Dong L., Dong H., Zhang Q., Zhao M., Yu L., Pang B., Chen Y. (2017). Graphene oxide modified polyamide reverse osmosis membranes with enhanced chlorine resistance. J. Membr. Sci..

[11] Liu J., Liu Y., Liu N., Han Y., Zhang X., Huang H., Lifshitz Y., Lee S.-T., Zhong J., Kang Z. (2015). Metal-free efficient photocatalyst for stable visible water splitting via a two-electron pathway. Science.

[12] Shen X., Zhang T., Xu P., Zhang L., Liu J., Chen Z. (2017). Growth of C3N4 nanosheets on carbon-fiber cloth as flexible and macroscale filter-membrane-shaped photocatalyst for degrading the flowing wastewater. Appl. Catal. B Environ..

[13] Sundar K.P., Kanmani S. (2020). Progression of Photocatalytic reactors and it’s comparison: A Review. Chem. Eng. Res. Des..

[14] Liu A.Y., Cohen M.L. (1989). Prediction of New Low Compressibility Solids. Science.

[15] Dong G., Zhang Y., Pan Q., Qiu J. (2014). A fantastic graphitic carbon nitride (g-C_3_N_4_) material: Electronic structure, photocatalytic and photoelectronic properties. J. Photochem. Photobiol. C Photochem. Rev..

[16] Wen J., Xie J., Chen X., Li X. (2017). A review on g-C_3_N_4_-based photocatalysts. Appl. Surf. Sci..

[17] Teter D.M., Hemley R.J. (1996). Low-Compressibility Carbon Nitrides. Science.

[18] Wang X., Maeda K., Thomas A., Takanabe K., Xin G., Carlsson J.M., Domen K., Antonietti M. (2009). A metal-free polymeric photocatalyst for hydrogen production from water under visible light. Nat. Mater..

[19] Safaei-Ghomi J., Akbarzadeh Z., Teymuri R. (2019). ZnS nanoparticlesimmobilized on graphitic carbon nitride as a recyclable and environmentally friendly catalyst for synthesis of 3-cinnamoyl coumarins. Res. Chem. Intermed..

[20] Wei B., Wang C., He Y., Ran G., Song Q. (2021). A novel FeS_2_@g-C_3_N_4_ composite with enhanced photo-Fenton catalytic activity for pollutant degradation. Compos. Commun..

[21] Kroke E., Schwarz M., Horath-Bordon E., Kroll P., Noll B., Norman A.D. (2002). Tri-s-triazine derivatives. Part I. From trichloro-tri-s-triazine to graphitic C_3_N_4_ structures. New J. Chem..

[22] Lukatskaya M.R., Mashtalir O., Ren C.E., Dall Y., Rozier P., Taberna P.L., Naguib M., Simon P., Barsoum M.W., Gogotsi Y. (2013). Cation intercalation and high volumetric capacitance of two-dimensional titanium carbide. Science.

[23] Ye M., Wang X., Liu E., Ye J., Wang D. (2018). Boosting the Photocatalytic Activity of P25 for Carbon Dioxide Reduction by using a Surface-Alkalinized Titanium Carbide MXene as Cocatalyst. ChemSusChem.

[24] Naguib M., Kurtoglu M., Presser V., Lu J., Niu J.J., Heon M., Hultman L., Gogotsi Y., Barsoum M.W. (2011). Two-Dimensional Nanocrystals Produced by Exfoliation of Ti_3_AlC_2_. Adv. Mater..

[25] Peng C., Wei P., Li X., Liu Y., Cao Y., Wang H., Yu H., Peng F., Zhang L., Zhang B. (2018). High efficiency photocatalytic hydrogen production over ternary Cu/TiO_2_@Ti_3_C_2_Tx enabled by low-work-function 2D titanium carbide. Nano Energy.

[26] Anasori B., Lukatskaya M.R., Gogotsi Y. (2017). 2D metal carbides and nitrides (MXenes) for energy storage. Nat. Rev. Mater..

[27] Liu W., Sun M., Ding Z., Gao B., Ding W. (2021). Ti_3_C_2_ MXene embellished g-C3N4 nanosheets for improving photocatalytic redox capacity. J. Alloys Compd..

[28] Zhuang Y., Liu Y., Meng X. (2019). Fabrication of TiO_2_ nanofibers/MXene Ti_3_C_2_ nanocomposites for photocatalytic H2 evolution by electrostatic self-assembly. Appl. Surf. Sci..

[29] Attanayake N.H., Abeyweera S.C., Thenuwara A.C., Anasori B., Gogotsi Y., Sun Y., Strongin D.R. (2018). Vertically aligned MoS_2_ on Ti_3_C_2_ (MXene) as an improved HER catalyst. J. Mater. Chem. A.

[30] Wang H., Wu Y., Yuan X., Zeng G., Zhou J., Wang X., Chew J.W. (2018). Clay-inspired MXene-based electrochemical devices and photo-electrocatalyst: State-of-the-art progresses and challenges. Adv. Mater..

[31] Huang K., Li C., Li H., Ren G., Wang L., Wang W., Meng X. (2020). Photocatalytic applications of two-dimensional Ti_3_C_2_ MXenes: A review. ACS Appl. Nano Mater..

[32] Ran J., Gao G., Li F.-T., Ma T.-Y., Du A., Qiao S.-Z. (2017). Ti_3_C_2_ MXene co-catalyst on metal sulfide photo-absorbers for enhanced visible-light photocatalytic hydrogen production. Nat. Commun..

[33] Jatoi A.S., Mubarak N.M., Hashmi Z., Solangi N.H., Karri R.R., Hua T.Y., Mazari S.A., Koduru J.R., Alfantazi A. (2023). New insights into MXene applications for sustainable environmental remediation. Chemosphere.

[34] Yin H., Yuan C., Lv H., Zhang K., Chen X., Zhang Y. (2022). Hierarchical Ti_3_C_2_ MXene/Zn_3_In_2_S_6_ schottky junction for efficient visible-light-driven Cr(VI) photoreduction. Ceram. Int..

[35] Hu J., Ding J., Zhong Q. (2021). Ultrathin 2D Ti_3_C_2_ MXene Co-catalyst anchored on porous g-C_3_N_4_ for enhanced photocatalytic CO_2_ reduction under visible-light irradiation. J. Colloid Interface Sci..

[36] Yang S., Wang K., Chen Q., Wu Y. (2024). Enhanced photocatalytic hydrogen production of S-scheme TiO_2_/g-C_3_N_4_ heterojunction loaded with single-atom Ni. J. Mater. Sci. Technol..

[37] Shi W.N., Fang W.X., Wang J.C., Qiao X., Wang B.B., Guo X.W. (2021). pH-controlled mechanism of photocatalytic RhB degradation over g-C_3_N_4_ under sunlight irradiation. Photochem. Photobiol. Sci..

[38] Le C., Chen T.Q., Liang T., Zhang P., MacMillan D.W.C. (2018). A radical approach to the copper oxidative addition problem: Trifluoromethylation of bromoarenes. Science.

[39] He H., Chen Y., Yang C., Yang L., Jiang Q., Huang H. (2022). Constructing 3D interweaved MXene/graphitic carbon nitride nanosheets/graphene nanoarchitectures for promoted electrocatalytic hydrogen evolution. J. Energy Chem..

[40] Li F., Liu Y.-L., Wang G.-G., Zhang H.-Y., Zhang B., Li G.-Z., Wu Z.-P., Dang L.-Y., Han J.-C. (2019). Few-layered Ti_3_C_2_Tx MXenes coupled with Fe_2_O_3_ nanorod arrays grown on carbon cloth as anodes for flexible asymmetric supercapacitors. J. Mater. Chem. A.

[41] He S., Zhan Y., Hu J., Zhang G., Zhao S., Feng Q., Yang W. (2020). Chemically stable two-dimensional MXene@UIO-66-(COOH)2 composite lamellar membrane for multi-component pollutant-oil-water emulsion separation. Compos. Part B Eng..

[42] Tu W., Liu Y., Chen M., Ma L., Li L., Yang B. (2022). Photocatalytic self-cleaning graphene oxide membrane coupled with carbon nitride and Ti_3_C_2_-Mxene for enhanced wastewater purification. Sep. Purif. Technol..

[43] Ren H.-T., Li D.-S., Cai C.-C., Li T.-T., Lou C.-W., Lin J.-H., Han X. (2023). Facile Photopolymerization of High-Molecular-Weight Polyaniline Composites Induced by g-C_3_N_4_ at Room Temperature for Trace Fe Ion Sensors. Macromolecules.

[44] Xiao R., Zhao C., Zou Z., Chen Z., Tian L., Xu H., Tang H., Liu Q., Lin Z., Yang X. (2020). In situ fabrication of 1D CdS nanorod/2D Ti_3_C_2_ MXene nanosheet Schottky heterojunction toward enhanced photocatalytic hydrogen evolution. Appl. Catal. B Environ..

[45] Zheng D., Pang C., Liu Y., Wang X. (2015). Shell-engineering of hollow g-C_3_N_4_ nanospheres via copolymerization for photocatalytic hydrogen evolution. Chem. Commun..

[46] Huang H.J., Yu D.S., Hu F., Huang S.C., Song J.N., Chen H.Y., Li L.L., Peng S.J. (2022). Clusters Induced Electron Redistribution to Tune Oxygen Reduction Activity of Transition Metal Single-Atom for Metal-Air Batteries. Angew. Chem. Int. Ed..

[47] Xu H., Xiao R., Huang J., Jiang Y., Zhao C., Yang X. (2021). In situ construction of protonated g-C_3_N_4_/Ti_3_C_2_ MXene Schottky heterojunctions for efficient photocatalytic hydrogen production. Chin. J. Catal..

[48] Li K., Lu X., Zhang Y., Liu K., Huang Y., Liu H. (2020). Bi_3_TaO_7_/Ti_3_C_2_ heterojunctions for enhanced photocatalytic removal of water-borne contaminants. Environ. Res..

[49] Li X., Bai Y., Shi X., Huang J.D., Zhang K., Wang R., Ye L.Q. (2021). Mesoporous g-C3N4/MXene (Ti_3_C_2_Tx) heterojunction as a 2D electronic charge transfer for efficient photocatalytic CO_2_ reduction. Appl. Surf. Sci..

[50] Tian X., Wu H., Hu X., Wang Z., Ren C., Cheng Z., Dou L., Lin Y.-W. (2022). Enhanced photocatalytic performance of ZnO/AgCl composites prepared by high-energy mechanical ball milling. New J. Chem..

[51] Zarezadeh S., Habibi-Yangjeh A., Mousavi M., Ghosh S. (2020). Novel ZnO/Ag_3_PO_4_/AgI photocatalysts: Preparation, characterization, and the excellent visible-light photocatalytic performances. Mater. Sci. Semicond. Process..

[52] Ding Q., Zou X., Ke J., Dong Y., Cui Y., Ma H. (2023). Enhanced artificial nitrogen fixation efficiency induced by construction of ternary TiO_2_/MIL-88A(Fe)/g-C_3_N_4_ Z-scheme heterojunction. J. Colloid Interface Sci..

[53] Feng C., Rong J., Zhang Y., Zheng X., Li X., Xu S., Li Z. (2023). An S-scheme CeO_2_/foveolate g-C_3_N_4_ composite with horseradish peroxidase activity for photo-enzyme synergistic catalytic degradation of phenanthrene. Appl. Catal. B Environ. Energy.

[54] Zhao Z., Zou Y., Liu P., Lai Z., Wen L., Jin Y. (2022). EIS equivalent circuit model prediction using interpretable machine learning and parameter identification using global optimization algorithms. Electrochim. Acta.

[55] Xu H., Li H., Sun G., Xia J., Wu C., Ye Z., Zhang Q. (2010). Photocatalytic activity of La_2_O_3_-modified silver vanadates catalyst for Rhodamine B dye degradation under visible light irradiation. Chem. Eng. J..

[56] Cui Y., Yang L., Meng M., Zhang Q., Li B., Wu Y., Zhang Y., Lang J., Li C. (2019). Facile preparation of antifouling g-C_3_N_4_/Ag_3_PO_4_ nanocomposite photocatalytic polyvinylidene fluoride membranes for effective removal of rhodamine B. Korean J. Chem. Eng..

[57] Huang J., Hu J., Shi Y., Zeng G., Cheng W., Yu H., Gu Y., Shi L., Yi K. (2019). Evaluation of self-cleaning and photocatalytic properties of modified g-C_3_N_4_ based PVDF membranes driven by visible light. J. Colloid Interface Sci..

[58] Issarapanacheewin S., Wetchakun K., Phanichphant S., Kangwansupamonkon W., Wetchakun N. (2016). Efficient photocatalytic degradation of Rhodamine B by a novel CeO_2_/Bi_2_WO_6_ composite film. Catal. Today.

[59] Kolesnyk I., Kujawa J., Bubela H., Konovalova V., Burban A., Cyganiuk A., Kujawski W. (2020). Photocatalytic properties of PVDF membranes modified with g-C_3_N_4_ in the process of Rhodamines decomposition. Sep. Purif. Technol..

[60] Guo Z., Zhang J., Liu H. (2016). Ultra-high Rhodamine B adsorption capacities from an aqueous solution by activated carbon derived from Phragmites australis doped with organic acid by phosphoric acid activation. RSC Adv..

[61] Wei Z., Spinney R., Ke R., Yang Z., Xiao R. (2016). Effect of pH on the sonochemical degradation of organic pollutants. Environ. Chem. Lett..

[62] Yue X., Liu Z., Zhang Q., Li X., Hao F., Wei J., Guo W. (2016). Oxidative degradation of Rhodamine B in aqueous solution using Fe/PANI nanoparticles in the presence of AQS serving as an electron shuttle. Desalination Water Treat..

[63] Fang S., Lv K., Li Q., Ye H., Du D., Li M. (2015). Effect of acid on the photocatalytic degradation of rhodamine B over g-C_3_N_4_. Appl. Surf. Sci..

[64] Wang Q., Zhou H., Qian J., Xue B., Du H., Hao D., Ji Y., Li Q. (2024). Ti3C2-assisted construction of Z-scheme MIL-88A(Fe)/Ti_3_C_2_/RF heterojunction: Multifunctional photocatalysis-in-situ-self-Fenton catalyst. J. Mater. Sci. Technol..

[65] Zhang Y., Zhou K., Yuan C., Lv H., Yin H., Fei Q., Xiao D., Zhang Y., Lau W. (2024). In-situ formation of SrTiO_3_/Ti_3_C_2_ MXene Schottky heterojunction for efficient photocatalytic hydrogen evolution. J. Colloid Interface Sci..

