# Supplementary material for: Carbon Nanofiber Membranes Loaded with MXene@g-C3N4: Preparation and Photocatalytic Property
Source: Nanomaterials (Basel). 2024 May 20;14(10):896. doi: 10.3390/nano14100896 (PMC11124281; doi:10.3390/nano14100896)
Supplement: Supplementary file 1 [file nanomaterials-14-00896-s001.zip › nanomaterials-3002445-supplementary.pdf]

Supporting Information

Carbon Nanofiber Membranes Loaded with MXene @ g-C_3N_4: Preparation and Photocatalytic Property

Ching-Wen Lou [1,2,3], Meng-Meng Xie [1], Yan-Dong Yang [1], Hong-Yang Wang [4], Zhi-Ke Wang [1], Lu Zhang [1,5], Chien-Teng Hsieh [6], Li-Yan Liu [1,5], Mei-Chen Lin [7,*] and Ting-Ting Li [1,5,*]

1 Innovation Platform of Intelligent and Energy-Saving Textiles, School of Textile Science and Engineering, Tiangong University, Tianjin 300387, China
2 Department of Bioinformatics and Medical Engineering, Asia University, Taichung 413305, Taiwan
3 Department of Medical Research, China Medical University Hospital, China Medical University, Taichung 404333, Taiwan
4 Tianjin Fire Science and Technology Research Institute of MEM, Tianjin 300381, China
5 Tianjin and Ministry of Education Key Laboratory for Advanced Textile Composite Materials, Tiangong University, Tianjin 300387, China
6 Department of Fashion Design and Merchandising, Shih Chien University, Kaohsiung 84550, Taiwan
7 Department of Biomedical Engineering, College of Biomedical Engineering, China Medical University, Taichung 404333, Taiwan
* Correspondence: lmc9363@mail.cmu.edu.tw (M.-C.L.); tingtingli@tiangong.edu.cn (T.-T.L.)

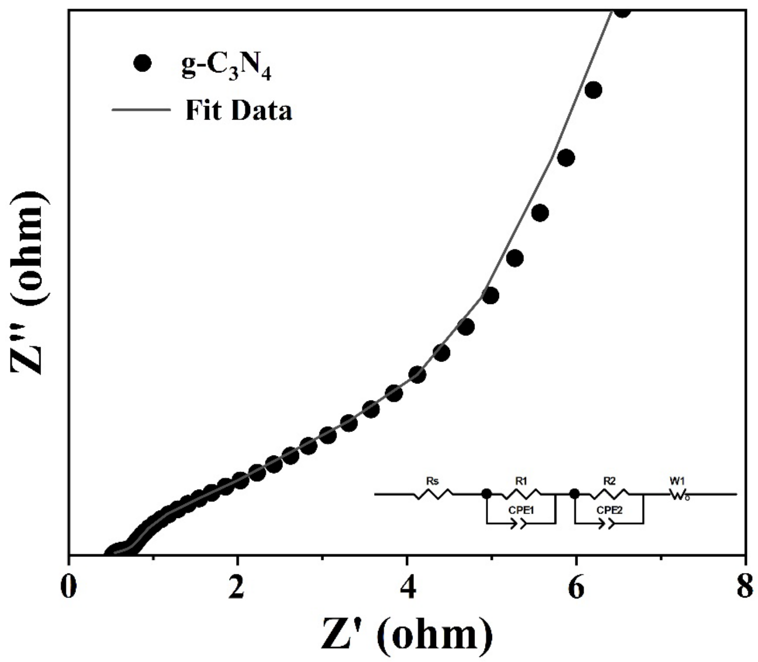

Figure S1. The Nyquist plots and corresponding circuits of g-C_3N_4.

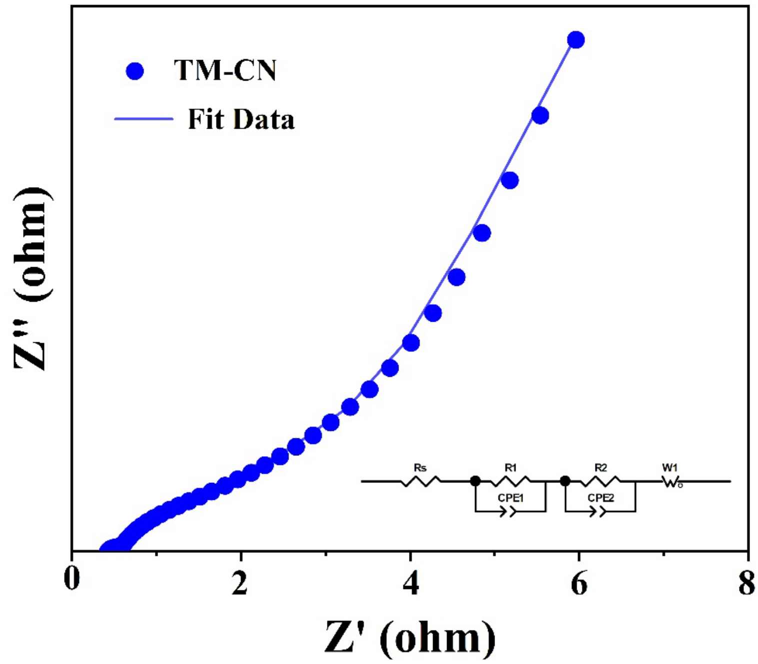

Figure S2. The Nyquist plots and corresponding circuits of TM-CN.

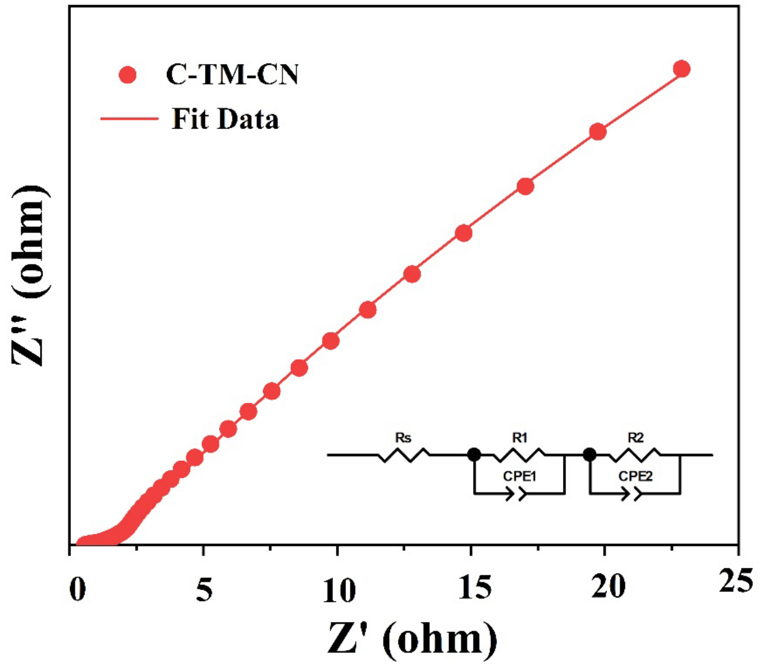

Figure S3. The Nyquist plots and corresponding circuits of C-TM-CN.
